# Reliability and validity of surface EMG assessments combined with isometric muscle strength testing in patients with abdominal rectus diastasis and asymptomatic controls

**DOI:** 10.1007/s10029-024-03076-y

**Published:** 2024-06-08

**Authors:** E. Swedenhammar, O. Wahlström, J. D. Brandt, K. Strigård, C. Häger, B. Stark, A. Nyberg

**Affiliations:** 1 Department of Molecular Medicine and Surgery, Karolinska Institutet, Stockholm, Sweden; 2 Department of Community Medicine and Rehabilitation, Umeå University, Umeå, Sweden; 3 Department of Surgical and Perioperative Sciences. Units: Surgery, Umeå University, Umeå, Sweden

**Keywords:** Abdominal rectus diastasis, Clinical investigation, Surface EMG, Reliability, Correlation

## Abstract

**Purpose:**

Patients with abdominal rectus diastasis (ARD) may have muscular functional impairments, but clinics lack appropriate objective assessment tools. The aim was to establish the relative and absolute reliability, and convergent validity, of muscular activity using Surface Electromyography (SEMG) during isometric abdominal muscle strength testing in patients with ARD and controls without ARD.

**Methods:**

Twenty-six patients with ARD were matched for age, sex and BMI with controls without ARD. Participants were tested twice during isometric muscular contractions using SEMG located on six abdominal sites. Mean amplitude, fatigue, and recruitment order were analyzed. Relative reliability was evaluated with Intraclass Correlation Coefficients (ICC), while absolute reliability was estimated by calculating the Standard Error of Measurement and Minimal Detectable Change. Convergent validity was addressed in relation to participant characteristics, functional ability, and symptoms.

**Results:**

Mean SEMG amplitude for all abdominal wall muscle contractions showed moderate to excellent relative test–retest reliability, with ICC values ranging from 0.46 to 0.97. In contrast, fatigue and recruitment order displayed poor to moderate relative reliability in both groups. Absolute reliability measures were generally high. A moderate to high convergent validity (ARD: rho-value 0.41–0.70; Controls: rho-value 0.41–0.75) was observed for mean amplitude in relation to a functional sit-to-stand test, abdominal circumference, BMI, back pain, and quality-of-life.

**Conclusions:**

The results of applying SEMG during isometric abdominal muscle support practicing the method in clinics, although additional development is needed with further standardization and more functional testing. Furthermore, the method demonstrates construct validity in patients with ARD and in age- and sex-matched controls.

## Introduction

Abdominal rectus diastasis (ARD) is a primary or secondary condition affecting both sexes. ARD can evolve following pregnancy, previous abdominal surgery, or high mass of intra-abdominal fat. It is more frequent in females and often related to hormonal changes during pregnancy [1–3]. When ARD is present, pain, discomfort, or impaired core stability during physical activity are often perceived [4–6].

During muscle activity of the abdominal wall, several muscles are engaged. Recruitment order of muscular activation is important [7], and women with ARD have shown significantly reduced muscle endurance and strength across different isometric abdominal muscular tests compared to females without ARD [8]. However, even though isometric tests of abdominal strength and endurance have excellent relative test–retest reliability in patients with large ventral hernias [9], the validity of these measurements could be questioned as only low/moderate correlations (r = − 0.34 to 0.50) between isometric abdominal muscle strength and the size of the ARD [9, 10]. They do not, for example, describe any significant associations to potentially relevant measurements such as the width of the ARD, abdominal circumference, or daily physical activity [10, 11]. Thus, an isometric abdominal muscle strength test might not be sufficient in clinical day-to-day work since the validity of these measurements is uncertain and additional objective assessment methods are warranted.

Surface electromyography (SEMG) is a valid method with excellent test–retest reliability to measure activity in m. obliquus externus and m. rectus abdominis in people with low back pain when performing abdominal drawing-in maneuvers to strengthen the deep abdominal muscles as well as in healthy individuals [12–15]. The potential benefit of combining SEMG measurements with isometric abdominal muscle tests in patients with ARD, as well as the psychometric properties of SEMG in this context, are yet to be determined. SEMG in combination with an isometric abdominal muscle test may provide additional value as it quantifies individual muscular function and renders information on activation patterns of abdominal wall muscles in patients with ARD.

The primary aim of this study was to determine the relative and absolute reliability of SEMG measurements of amplitude, a fatigue estimate, and recruitment order during isometric abdominal muscle strength testing in patients with ARD and in a control group matched for age, BMI, and sex.

The secondary aim was to evaluate the potential added value of SEMG measurements to an isometric abdominal strength test by determining and comparing the convergent validity between SEMG measurements, isometric muscle strength, and other clinically relevant measures, including a functional sit-to-stand test, the width of the ARD, bodily pain, quality-of-life (QoL), body perception, and abdominal- and/or back pain. The recruitment order of the muscles was also compared descriptively between the groups.

## Method and materials

### Study design and participants

This experimental cross-sectional cohort study comprises test-retests in patients with ARD and a group of matched controls without ARD according to “Guidelines for Reporting Reliability and Agreement Studies” [16]. Patients with ARD, and a request for surgical repair because of functional disabilities, referred to either the Centre of Hand and Plastic Surgery Department or to the Centre of Surgery at Umeå University Hospital were invited to participate. Inclusion criteria were ARD width ≥ 3 cm at either of the two measured abdominal sites, namely the half measurement between the umbilicus and the xiphoidal process and the umbilicus and the symphysis, age over 18 years, and an understanding of the written and oral information regarding consent to participate. Exclusion criteria were previous large abdominal or back surgery, ongoing smoking, pregnancy, and less than 2 years since any bariatric surgery. A control group without ARD matched for age, BMI, and sex was also recruited by convenience sampling among staff, acquaintances, and by advertising. The Declaration of Helsinki principles of ethical standards were followed, and written informed consents were obtained. The study was approved by the Regional Ethical Review Board (Dnr: 2019-05348, 2020-04335).

### Procedure

Participants were investigated twice, with one week in between, at the Umeå Movement and Exercise Laboratory, Department of Community Medicine and Rehabilitation (Umeå, Sweden). Each test of about 45 min was performed by the same investigator (OW) including oral instructions, measurements and placement of SEMG electrodes. Demographics in terms of sex, body weight, body height, previous smoking habits, age, earlier surgical procedures, previous weight loss, pain in the abdomen and/or back were collected with open questions as well as information about potential difficulties when performing physical activity or sport.

### Clinical investigation

Participants were placed on a treatment table in a supine, relaxed position. The circumference of the abdomen at the umbilical level was determined with a tape measure. The medial border of the m. rectus abdominis was palpated while the participant raised their head and neck, and the width of ARD was measured at two locations of the Linea alba as described above.

### Isometric abdominal muscle strength

Isometric abdominal muscle strength was measured using a computerized stationary dynamometer (Biodex System Pro 4 Biodex Medical Systems, Shirley, New York, NY, USA) [17]. Muscular strength was measured in Newton meter (Nm), and the highest peak torque of five isometric contractions was chosen, presented in absolute values. All patients started the test with hips and knees in 90-degree flexion (zero position). Upper body and lower extremities were fixated with straps. A pillow was placed below the scapular area, permitting a 10-degree thoracic flexion. The participants were instructed to perform a muscular contraction when a sound signal was given. Before starting the tests, all participants warmed up with five repetitions of flexion with increasing intensity, followed by a mandatory 2-min rest. Five seconds rest between every isometric contraction was also included. Strong and equal verbal encouragement was given to all participants.

### Surface EMG

**Fig. 1:**
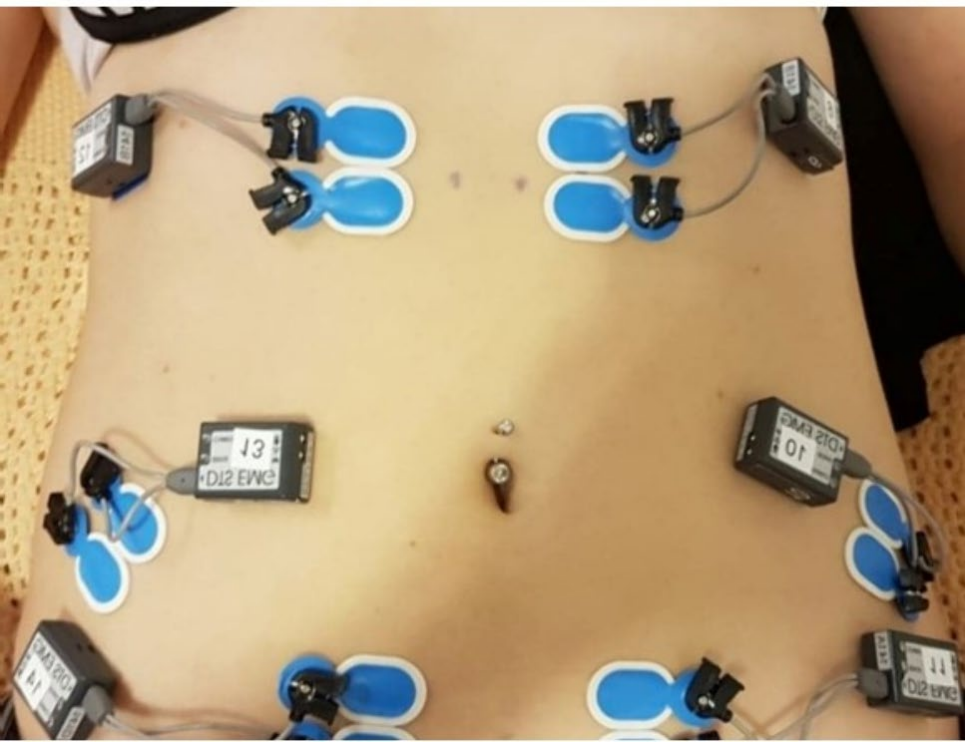
Placement of SEMG electrodes. *SEMG* surface electromyography

During the isometric abdominal muscular strength test, we collected SEMG with a sampling rate of 1500 Hz on a TeleMyo direct transmission system (model 542 DTS EMG sensor, Noraxon USA Inc., US) using silver-silver chloride, pre-gelled bipolar surface electrodes (Ambu® BlueSensor N, Ballerup, Denmark). After shaving, scrubbing, and cleaning the skin surface with isopropyl alcohol, electrodes were placed on the muscle belly with an inter-electrode distance of 20 mm following the SENIAM guidelines [18]. Six pairs of electrodes were placed in a standardized design in line with bilateral anatomical landmarks, as described by Gilleard et al. [19]; see Fig. 1. Spots half-distance between the symphysis and the umbilicus and half-distance between the xiphoid process and the umbilicus were marked, 3 cm laterally from the medial margin of the muscles. These four sites defined the dimensions of the rectus muscles at each body side, and both upper and lower bellies. The external oblique muscle site was located at half-distance between the arcus costalis and the anterior superior iliac spine. The electrodes were placed across the alignment of the muscular fibres.

All measurements were photographically documented to ensure the same placement at the time of retest, and the electrodes’ placements were also accentuated with a surgical marker pen. Information was recorded regarding amplitude, symmetry of the muscles, timing of activation and peak torque for maximal contractions. SEMG data were analyzed manually by one investigator (JDB) with MyoResearch XP Master Edition 1.07.63 software. The signal was first processed with an algorithm to reduce heart rate interference, and then band-pass filtered (10–500 Hz) to reduce noise. Lastly, a moving RMS filter with a window size of 20 ms was used to rectify and smoothen the signal. Data were rectified, i.e., the negative portion of the signals was converted to positive values. This facilitated detection of the onset and offset of muscular activity. The task was mainly restrained to prevent the occurence of large movements that could potentially  cause movement artefacts. Further, we used wireless sensors (no cables), that are less sensitive to disturbances. The filtering of the SEMG signals, the thorough process with manual inspection for artefacts and correct event settings ensured quality. We set markers at µV cutoff values for "Rise" and "Fall" events using the MR-XP1.07 Master Edition software. The markers, confirmed by visual inspection and adjusted if necessary, indicated which muscle was activated first. Initial thresholds were set at 15 µV; if the automatised marker setting according to the algorithm was inadequate, adjustments were made, lowering thresholds to 10 µV or 5 µV for weak signals, or for stronger signals with increments of 5 µV, ranging from 20 to 45 µV. If initial marker placements were inaccurate or affected by baseline disturbances, manual corrections involved deleting and resetting markers to appropriate values. This process ensured that the markers were accurately set to represent the actual muscle activation order, and any significant potential disturbances such as or artefacts were accounted for by adjusting the threshold values as needed. Markers were placed at every onset of bilateral muscular contraction. Mean amplitude was calculated from each of the five contractions and presented in microvolts (µV). For muscular contractions, frequency-based analysis parameters display time domain changes due to muscular fatigue [20]. Because of the recruitment of motor units, the frequency-based mean or median frequency of the total power spectrum usually show a decrease over contraction time. We therefore calculated a fatigue index, defined as a slope determined by the decrease of the mean frequency (Hz) of the combined action potentials during each muscle contraction. The recruitment order of the six muscles recorded was ranked from one to six depending on which muscle was first activated and the hereinafter following muscles.

### A modified five-time sit-to-stand test (5STS20)

Patients with ARD report difficulties standing up, particularly from a low chair [21]. The original 5STS was therefore modified [22] for the current purpose, and participants performed a sit-to-stand test in which a standardized chair height of 20 cm was applied. To optimize test–retest conditions, the position of the feet’s placement was standardized by measuring the distance between the metatarsophalangeal joints and that from the heel to the chair legs. Participants held their arms across the chest during the tests and familiarized themselves with the procedure before performing five repetitions of sit-to-stand as fast as possible. The time duration of the tests was registered in seconds. If the participant could not stand up, the test was terminated.

### Questionnaires

Four different questionnaires were completed at the time of retests: the Ventral Hernia Pain Questionnaire (VHPQ) [23], the Body Questionnaire (BODY-Q) [24], the Short Form 36 questionnaire (SF-36) [25], and the Roland Morris Disability Questionnaire (RMDQ) [26]. VHPQ is an established, validated questionnaire to evaluate the duration and level of pain during daily activities such as standing, walking, physical activities, and driving. It also evaluates pain before and after surgery plus patients´ satisfaction with the surgical procedure [23]. Body-Q is a validated questionnaire used to study the patient’s perception of their body after surgery or weight loss. It consists of modules of different body parts and general emotions regarding the body, and the form is validated to exert the part of the body that is assessed. Participants do not have to answer all parts of the questionnaire; only parts relevant to the study may be selected [24]. SF-36 is a well-known validated questionnaire to evaluate quality-of-life, both physical and emotional. It is designed to create health scores in both mental and physical dimensions. These dimensions can also be summarized in two-component scores. The results can be compared to a validated Swedish normal population matched for age and gender [25, 27]. The RMDQ survey consists of 24 yes- or no-questions that represent physical functions that might be associated with back pain [26].

### Statistics

All statistics were calculated using IBM^®^ SPSS^®^ Statistics, version 28.0.1.1. A total of 26 individuals with ARD were needed to detect an intraclass correlation coefficient (ICC) > 0.7 with a confidence interval of 0.4 based on the results from previous pilot testing on timing and amplitude measured with SEMG during isometric abdominal strength testing [28]. With a sample size of 26 individuals, a correlation coefficient of 0.5 between SEMG measurements, isometric abdominal muscle strength, width of the ARD, bodily pain, quality-of-life, body perception, and abdominal- and/or back pain could be detected. A correlation coefficient of 0.5 was considered the lowest correlation of interest.

Evaluation of relative and absolute test–retest reliability was conducted by testing the timing of activation of the different muscles (bilateral rectus muscle upper/lower and externus obliquus muscles), fatigue, and mean amplitude. Relative reliability was evaluated with ICC two-way mixed–absolute agreement with 95% confidence interval and was graded according to Koo et al. [29]. Cut-off reliability values of ICC were classified as: < 0.5 poor, 0.5–0.75 moderate, 0.76–0.9 good, > 0.9 excellent. The mean ICC value was used to determine the strength of the correlations in relation to the used cut-offs. Absolute reliability was assessed by calculating the minimal standard error of measurement (SEM) and the minimum detectable change (MDC) [30].

Convergent validity was evaluated with Spearman’s rank correlation coefficient (rho) for amplitude, recruitment order, and fatigue with rho values in the range of 0–1. To present a visual representation of the data, a heatmap was provided for both groups. Heat maps show relationships and highlight correlations between the different variables. The color scheme ranged from -1 for negative correlations to + 1 for positive correlations. Only variables with a mean ICC > 0.7 in accordance with our sample size estimation were assessed for convergent validity. Demographics were presented as the median and interquartile range (IQR 25–75). When comparing outcomes between the groups, the values from the first test were used. The Mann–Whitney U-test was used for continuous variables and the chi-square test was applied for categorical variables. *p* values < 0.05 were considered significant.

## Results

**Fig. 2:**
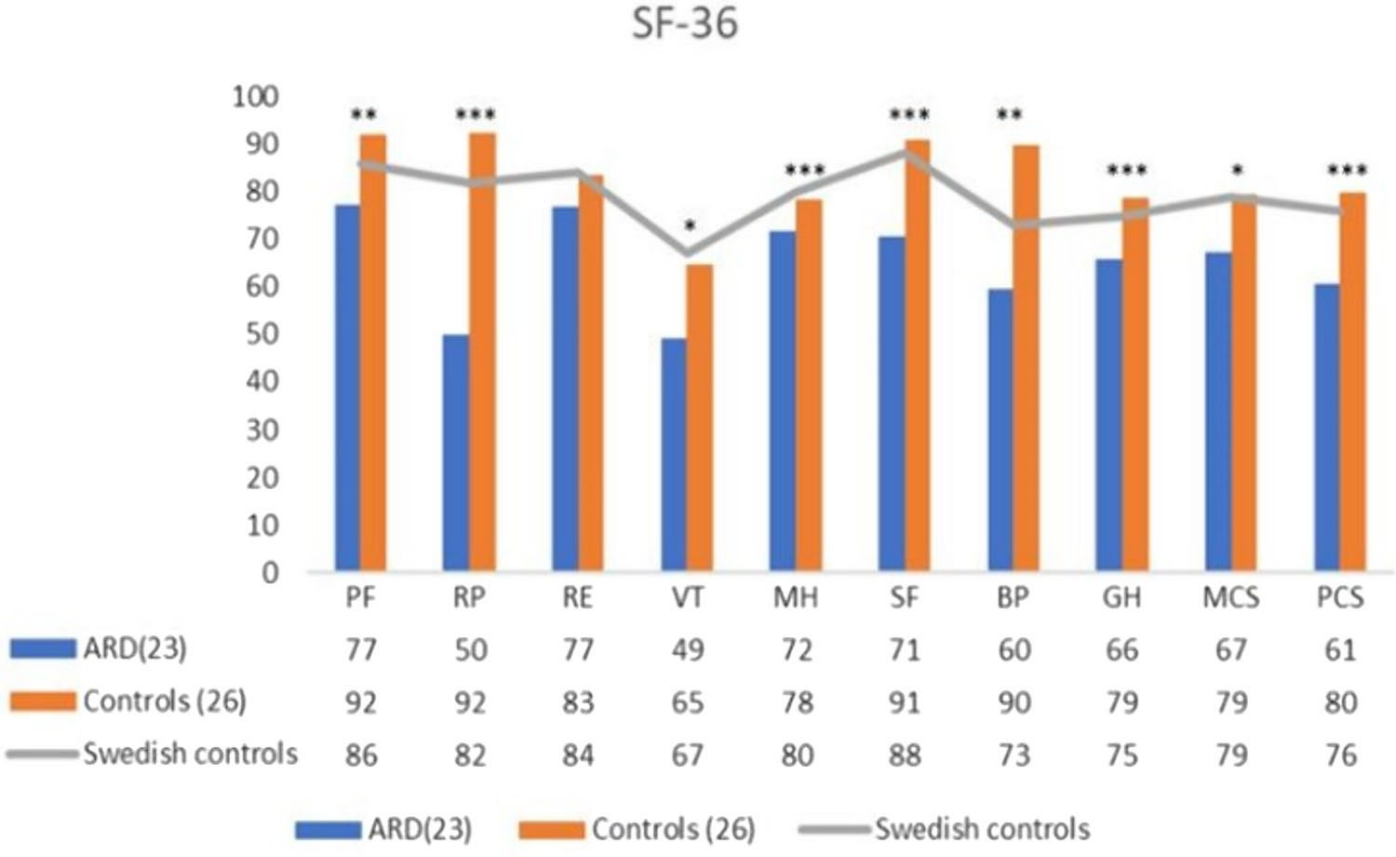
SF-36. Results from SF-36. The higher the number, the better the self-reported health-related quality-of-life. Significant differences *< 0.05. **< 0.01. ***< 0.001. PF = physical function, RP = role physical, RE = role emotional, VT = vitality, MH = mental health, SF = social function, BP = bodily pain, GH = general health, MCS = mental score component (composed from GH, VT, SF, RE, MH). PSC = physical score component (composed from GH, VT, SF, PF, RP, BP). The Swedish controls are reference values [27]

Twenty-six patients with ARD and twenty-six controls without ARD were included. Two individuals in the ARD group did not complete the SF-36 and VHPQ questionnaires. Participant characteristics are presented in Table 1 and Fig. 2. In brief, no differences were observed between the groups regarding sex, age, BMI, or abdominal circumference. However, participants with ARD reported significantly more general pain, back pain, and pain during various daily life activities, worse abdomen appearance, physical function, self-perceived body image, and lower quality-of-life. Patients with ARD scored lower on all SF-36 scales compared with the Swedish normative reference for females [27]. The control group was significantly stronger regarding isometric muscle contraction (ARD group 105.5 Nm, control group 121.6 Nm, *p* = 0.040).

**Table 1:** Participant characteristics

Anthropometrics	ARD, n = 26	Controls, n = 26	*p*
Sex female, N (%)	20 (77)	20 (77)	1
Age (years), median (IQR)	44 (39–55)	45 (37–57)	0.869
BMI (kg/m^2^), median (IQR)	23.4 (21.7–31.4)	24.0 (21.4–28.3)	0.784
Previous abdominal surgery, Yes N (%)	12 (46.2)	11 (42.1)	0.780
Abdominal circumference (cm), median (IQR)	81.5 (76.8–101.8)	83.0 (74.5–95.5)	0.591
Upper ARD (cm), median (IQR)	3.75 (3.00–4.75)***	0.00 (0.00–0.00)	< 0.001
Lower ARD (cm), median (IQR)	3.00 (0.00–4.00)***	0.00 (0.00–0.00)	< 0.001
*Pain measurements*			
Backpain, Yes N (%)	18 (69.0)**	8 (30.8)	0.006
Abdominal pain, Yes N (%)	15 (56.0)***	3 (11.5)	< 0.001
Pain when standing up, Yes N (%)	4 (15.4)	1 (3.8)	0.158
Pain during physical activity, Yes N (%)	17 (65.4)***	2 (7.7)	< 0.001
RMDQ (points), median (IQR)	3 (0.00–8.50)**	0 (0–1)	0.003
VHPQ pain right now ⩽ 1, Yes N (%)	14 (54.0)***	26 (100.0)	< 0.001
VHPQ pain right now > 1, Yes N (%)	10 (40.0)***	0 (0.0)	< 0.001
VHPQ pain when rising from chair, Yes N (%)	4 (17.0)*	0 (0.0)	0.020
VHPQ pain when sitting, Yes N (%)	2 (8.0)	0 (0.0)	0.103
VHPQ pain when standing, Yes N (%)	5 (21.0)*	0 (0.0)	0.020
VHPQ pain when driving car, Yes N (%)	1 (4.0)	0 (0.0)	0.480
VHPQ pain when performing sports, Yes N (%)	13 (54.0)***	0 (0.0)	< 0.001
*Appearance, function and symptoms*			
BODY-Q abdomen appearance (points), median (IQR)	14.5 (8.0–20.3)**	21.0 (15.0–22.3)	0.003
BODY-Q physical function (points), median (IQR)	24.0 (18.5–28.0)*	27.0 (25.5–28.0)	0.017
BODY-Q body image (points), median (IQR)	17.0 (13.0–23.0)*	21.0 (19.8–24.0)	0.046
5STS20 (seconds), median (IQR)	10.4 (9.1–12.2)*	8.8 (5.5–17.9)	0.014

Median and Interquartile range (IQR—q1–q3) are shown for continuous variables. Number (and percentage) of yes for categorical variables are presented. The higher the score in RMDQ, the more pain is present. In BODY-Q, the higher score describes a better self-reported conception of body image, the appearance of the abdomen, and physical function. VHPQ pain right now ⩽ 1, includes no pain (0) and/or pain that is easily ignored. Pain right now > 1 cannot be ignored. Significance levels *< 0.05. **< 0.01. ***< 0.001

### Relative and absolute test–retest reliability

**Fig. 3:**
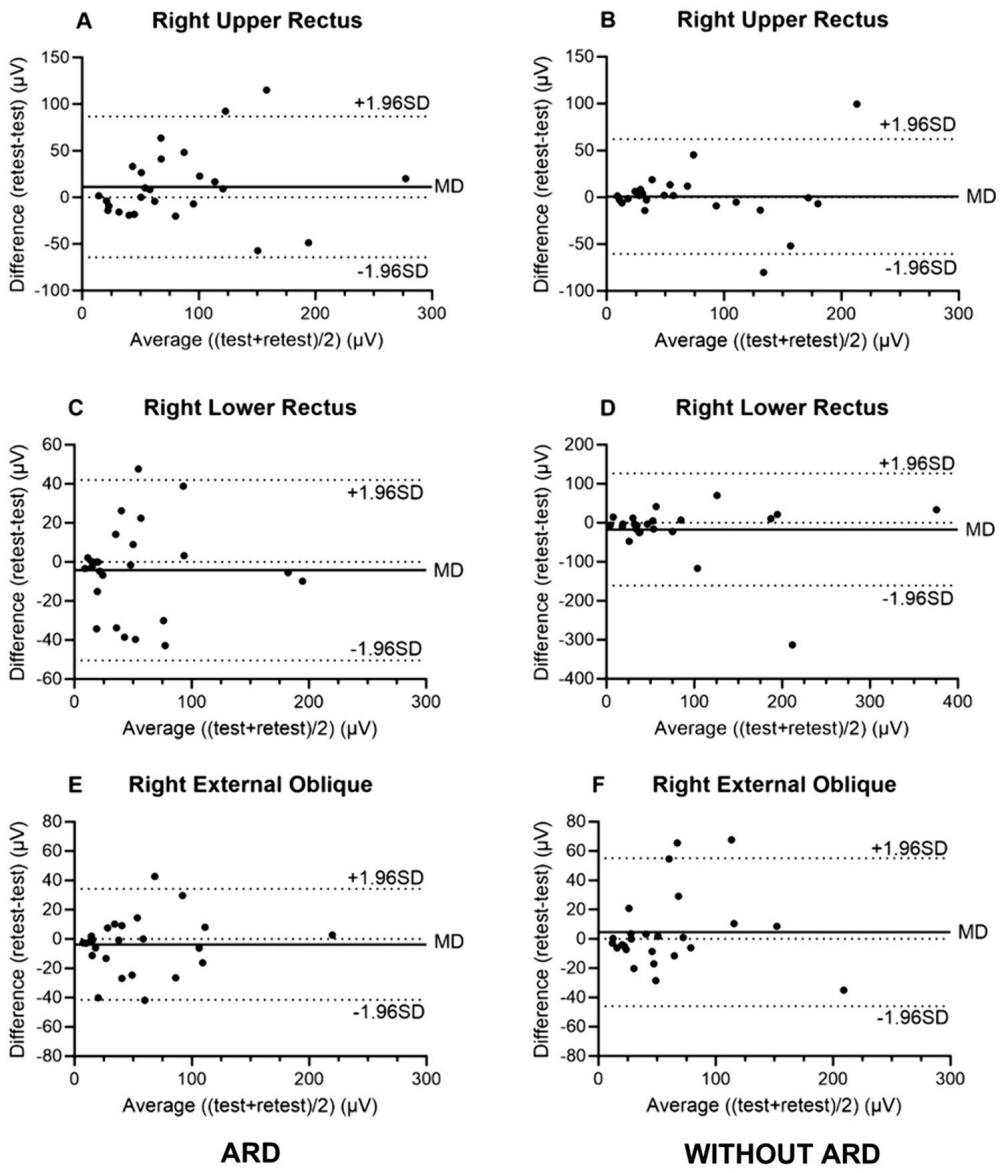
**A**–**F** Bland Altman plots for Mean amplitude on the right side of the abdomen. Bland Altman plots for the Mean Amplitude on the right side of the abdomen. ARD patients to the left, control group to the right. Each dot represents one participant’s value in relationship to the entire groups mean difference in µV. MD represents the mean of differences, and the upper and lower lines represent the 95% limits of agreement, here presented as ± 1.96 SD. A: ARD upper rectus right, B: control upper rectus right, C: ARD lower rectus right, D: control lower rectus right, E: ARD obliquus externus right, F: control obliquus externus right

**Fig. 4:**
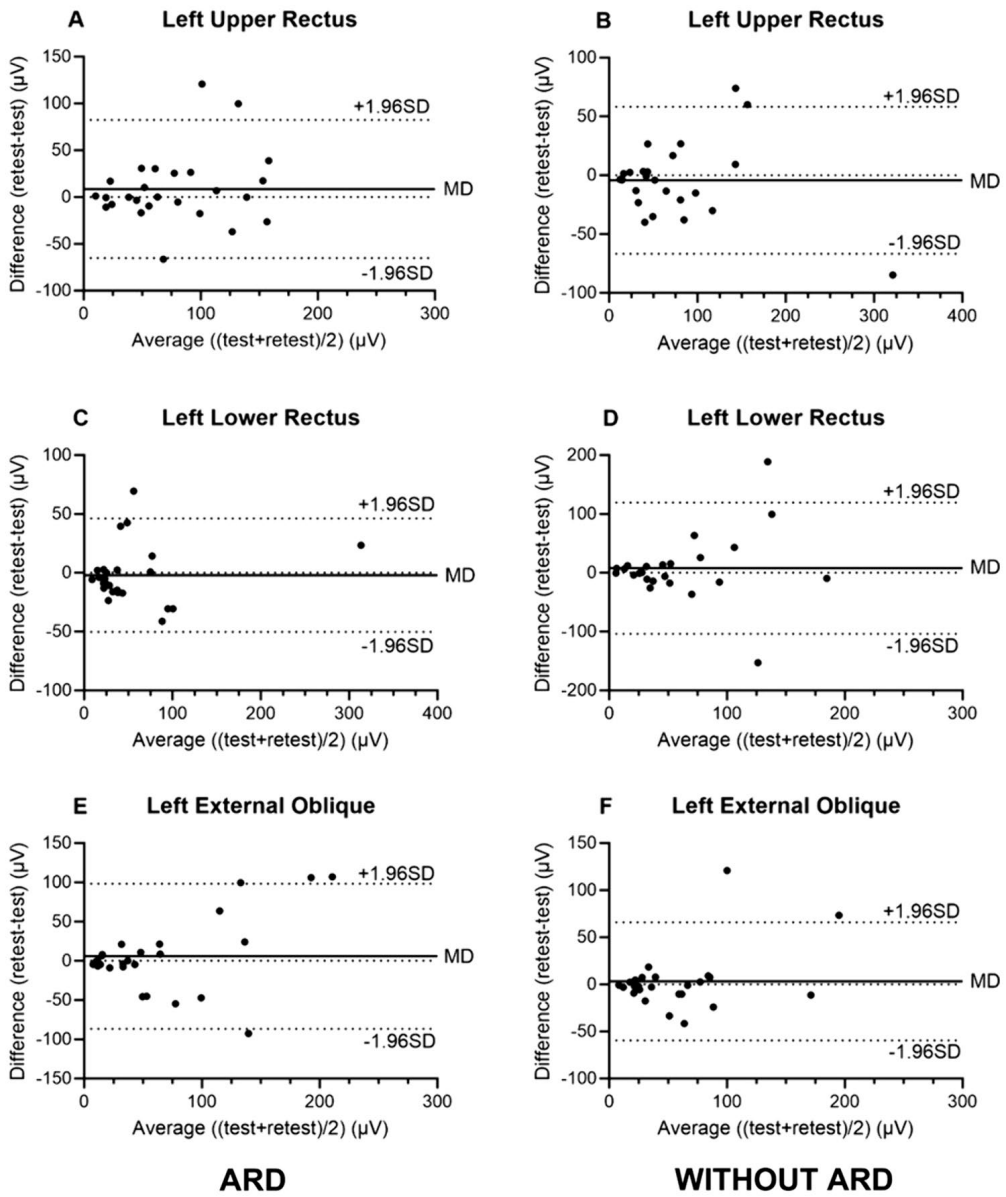
**A**–**F** Bland Altman plots for mean amplitude on the left side of the abdomen. Bland Altman plots for the mean amplitude on the right side of the abdomen. ARD patients to the left, control group to the right. Each dot represents one participant’s value in relationship to the entire groups mean difference in µV. MD represents the mean of differences, and the upper and lower lines represent the 95% limits of agreement, here presented as ± 1.96 SD. A: ARD upper rectus left, B: control upper rectus left, C: ARD lower rectus left, D: control lower rectus left, E: ARD obliquus externus left, F: control obliquus externus left

Relative reliability was poor to excellent in the isometric abdominal muscle strength test in the ARD group (mean ICC 0.642 to 0.915), but lower in the control group (mean ICC 0.390 to 0.840). In patients with ARD, SEMG measurements ranged from poor to excellent reliability, with amplitude being the most reliable, demonstrating moderate to excellent relative test–retest reliability across muscles (mean ICC 0.712–0.922). Measurements of absolute reliability for amplitude ranged from SEM 17.7–32.8 µV, and MDC 37.2–90.9 µV. Amplitude also demonstrated the highest relative reliability in the control group, still ranging from poor to good for different muscles (mean ICC 0.467–0.892) and comparable absolute reliability to the ARD group (SEM: 18.0–50.1 µV, MDC 50.0–138.9 µV), although this varied across muscles. Notably, relative reliability for recruitment order was poor to good in both patients with ARD (mean ICC: 0.402–0.609) and in the control group (mean ICC 0.373–0.579), while the relative reliability for decline in Fatigue measurement was generally poor (ARD: ICC 0.113–0.524, Control: ICC − 0.184 to 0.645). SEM and MDC values were generally high for recruitment order and Fatigue among patients with ARD and in the control group, respectively (Tables 2 and 3), indicating lower absolute reliability. Bland Altman plots for Amplitude are shown in  Figs. 3, 4.

**Table 2:** Relative and absolute test–retest reliability among patients with ARD

	Test	Retest	MD	Relative reliability	Absolute reliability	
				ICC (95%CI)	SEM	MDC
*Muscle strength* (Nm)						
Isometric muscle contraction	105.5 (33.5)	100.6 (36.2)	4.9	0.820 (0.642–0.915)	14.7	40.7
*Amplitude* (µV)						
Upper rectus right	77.2 (61.3)	88.4 (66.4)	11.2	0.811 (0.625–0.910)	27.6	76.5
Lower rectus right	54.4 (49.0)	49.3 (48.7)	5.0	0.886 (0.761–0.948)	17.7	49.1
External oblique right	55.3 (47.4)	50.7 (49.0)	4.6	0.921 (0.832–0.964)	13.4	37.2
Upper rectus left	72.9 (46.1)	81.5 (53.3)	8.6	0.712 (0.460–0.859)	26.5	73.5
Lower rectus left	53.8 (59.7)	50.2 (62.3)	3.6	0.922 (0.832–0.965)	16.9	46.8
External oblique left	61.3 (50.6)	67.1 (72.1)	5.8	0.718 (0.464–0.863)	32.8	90.9
*Fatigue* (Hz)						
Upper rectus right	− 1.4 (2.2)	− 2.2 (2.3)	0.79	0.524 (0.179–0.754)	1.6	4.4
Lower rectus right	− 1.0 (1.3)	− 0.5 (1.5)	0.48	0.113 (− 0.280 to 0.474)	1.3	3.7
External oblique right	− 0.5 (1.8)	− 1.0 (2.3)	0.48	0.187 (− 0.209 to 0.530)	1.8	5.1
Upper rectus left	− 0.3 (4.1)	− 1.6 (3.3)	1.24	0.303 (− 0.088 to 0.613)	3.1	8.6
Lower rectus left	− 1.2 (1.3)	− 0.9 (1.1)	0.36	0.364 (− 0.019 to 0.654)	1.0	2.6
External oblique left	− 0.7 (1.3)	− 0.9 (2.5)	0.18	0.260 (− 0.134 to 0.583)	1.7	4.6
*Recruitment order*						
Upper rectus right	3.6 (1.2)	3.4 (1.1)	0.2	0.590 (0.269–0.793)	0.7	2.0
Lower rectus right	3.3 (1.0)	3.3 (1.0)	0.0	0.428 (0.056–0.696)	0.8	2.1
External oblique right	3.5 (1.2)	3.9 (1.3)	0.4	0.329 (− 0.060 to 0.631)	1.0	2.9
Upper rectus left	3.9 (1.1)	3.7 (1.2)	0.3	0.534 (0.192–0.760)	0.8	2.1
Lower rectus left	3.5 (1.2)	3.4 (1.2)	0.1	0.402 (0.025–0.679)	0.9	2.5
External oblique left	3.2 (1.5)	3.4 (1.4)	0.2	0.609 (0.297–0.803)	0.9	2.4

The muscles are presented for each placement of the electrodes. The results are presented in mean and standard deviation (SD). SEMG is presented as mean amplitude, fatigue and recruitment order. MD = difference between means, ICC = intraclass correlation coefficient, CI = confidence interval, SEM = standard error of measurement, MDC = minimal detectable change. Significance level 0.05. Cut-off values of ICC as < 0.5 poor; 0.5–0.75 moderate; 0.76–0.9 good; > 0.9 excellent reliability was used [29]

**Table 3:** Relative and absolute test–retest reliability in the control group without ARD

	Test	Retest	MD	Relative reliability	Absolute reliability	
				ICC (95%CI)	SEM	MDC
*Muscle strength* (Nm)						
Isometric muscle contraction	121.6 (31.0)	122.3 (30.5)	0.7	0.670 (0.390–0.840)	17.5	48.6
*Amplitude* (µV)						
Upper rectus right	73.0 (62.1)	70.8 (61.8)	2.2	0.876 (0.735–0.944)	21.6	59.9
Lower rectus right	88.9 (100.2)	68.3 (84.9)	20.6	0.703 (0.417–0.862)	50.1	138.9
External oblique right	55.9 (47.9)	58.7 (49.4)	2.8	0.860 (0.709–0.936)	18.0	50.0
Upper rectus left	74.8 (71.3)	68.8 (65.0)	5.9	0.892 (0.771–0.951)	22.2	61.5
Lower rectus left	55.2 (50.1)	61.8 (59.0)	6.6	0.467 (0.096–0.724)	22.4	62.1
External oblique left	55.4 (42.8)	56.8 (54.4)	1.4	0.787 (0.575–0.900)	39.7	110.0
*Fatigue* (Hz)						
Upper rectus right	− 2.1 (2.2)	− 1.5 (3.2)	0.6	0.618 (0.302–0.811)	1.7	4.7
Lower rectus right	− 1.3 (0.9)	− 2.0 (3.0)	0.7	− 0.184 (− 0.534 to 0.220)^¥^		
External oblique right	− 1.6 (2.5)	− 0.8 (3.2)	0.8	− 0.610 (− 0.807 to 0.290)^¥^		
Upper rectus left	− 2.0 (2.7)	− 1.4 (2.7)	0.6	0.645 (0.342–0.826)	1.6	4.4
Lower rectus left	− 1.0 (3.3)	− 1.4 (1.4)	0.4	− 0.218 (− 0.559 to 0.186)^¥^		
External oblique left	− 1.1 (2.1)	− 0.9 (1.9)	0.2	0.142 (− 0.261 to 0.502)	1.8	5.1
*Recruitment order*						
Upper rectus right	3.8 (1.2)	3.8 (1.1)	0.1	0.373 (− 0.018 to 0.665)	0.9	2.5
Lower rectus right	3.0 (1.1)	3.1 (0.9)	0.1	0.479 (0.111–0.731)	0.7	2.0
External oblique right	3.6 (1.3)	3.5 (1.3)	0.1	0.464 (0.092–0.722)	0.9	2.5
Upper rectus left	3.6 (1.1)	3.9 (1.2)	0.3	0.445 (0.069–0.711)	0.9	2.4
Lower rectus left	3.5 (0.8)	3.4 (1.0)	0.1	0.579 (0.247–0.790)	0.6	1.6
External oblique left	3.5 (1.2)	3.4 (1.3)	0.2	0.368 (− 0.024 to 0.662)	1.0	2.3

The muscles are presented for each placement of the electrodes. The results are presented in mean and standard deviation (SD). SEMG is presented as mean amplitude. Fatigue and recruitment order. MD = difference between means, ICC = intraclass correlation coefficient, CI = confidence interval, SEM = standard error of measurement, MDC = minimal detectable change. Significance level 0.05. ^¥^When ICC value was negative. No further test for absolute reliability was tried. Cut-off values of ICC as < 0.5 poor; 0.5–0.75 moderate; 0.76–0.9 good; > 0.9 excellent reliability was used [29]

### Convergent validity

The potential added value of SEMG to an isometric abdominal strength test was determined by exploring the convergent validity of the SEMG measurements with acceptable relative reliability defined as ICC > 0.7 based on the pilot testing used for the sample size calculation. Thus, convergent validity was only explored for amplitude. In patients with ARD, significant and moderate to very large negative correlations (rho = − 0.414 to − 0.703) were observed between amplitude and BMI, back pain, abdominal circumference, upper ARD width, and sit-to-stand performance, while moderate positive correlations (rho = 0.428–0.440) were seen with self-perceived body image. Notably, isometric abdominal strength was only negatively correlated to back pain (rho = − 0.535) and positively correlated to the physical component summary of SF-36 (rho = 0.497). No significant correlations were observed between isometric abdominal strength and BMI, back pain, abdominal circumference, upper ARD width, or sit-to-stand performance (Table 4). In the control group, a similar pattern was observed. For example, moderate to very large negative correlations (rho = − 0.408 to − 0.753) between SEMG amplitude measurements and BMI, abdominal circumference, and sit-to-stand performance were demonstrated, while the isometric abdominal strength test was only correlated to age (rho = − 0.417) (Table 5).

**Table 4:**
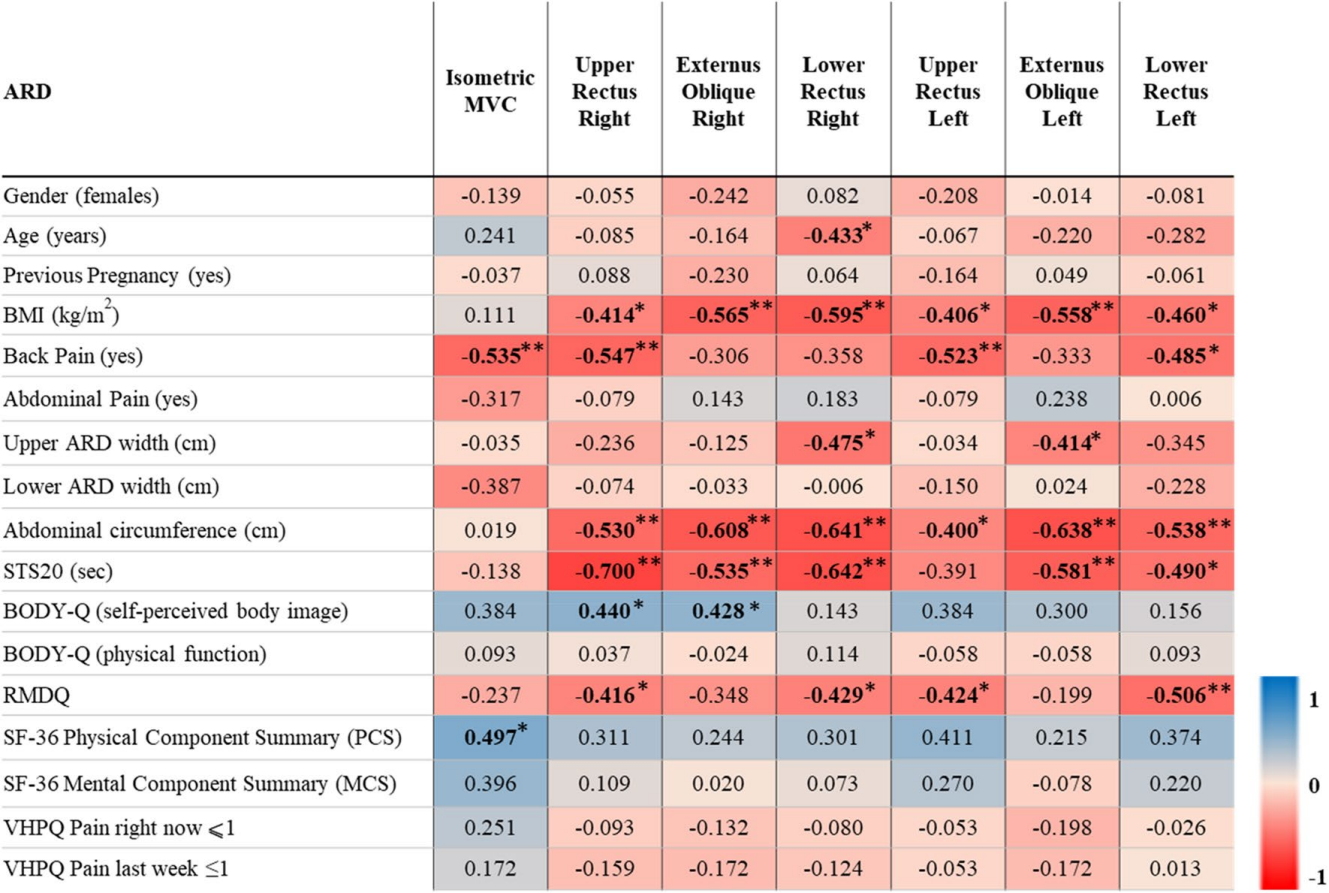
Convergent validity of SEMG amplitude data among patients with ARD

Spearman rank correlations (rho) between mean amplitude of SEMG and other variables. A correlation of 0.5 was considered the smallest correlation of interest. Cut-off values of 0.1–0.29 small, 0.3–0.49 moderate, 0.5–0.69 large, 0.7–0.89 very large and 0.9–1.0 extremely large were used to determine the strength of the correlations (28). To better show the correlations, red represents rho-values − 1 and blue 1

**Correlation is significant at the 0.01 level (2-tailed)

*Correlation is significant at the 0.05 level (2-tailed)

**Table 5:**
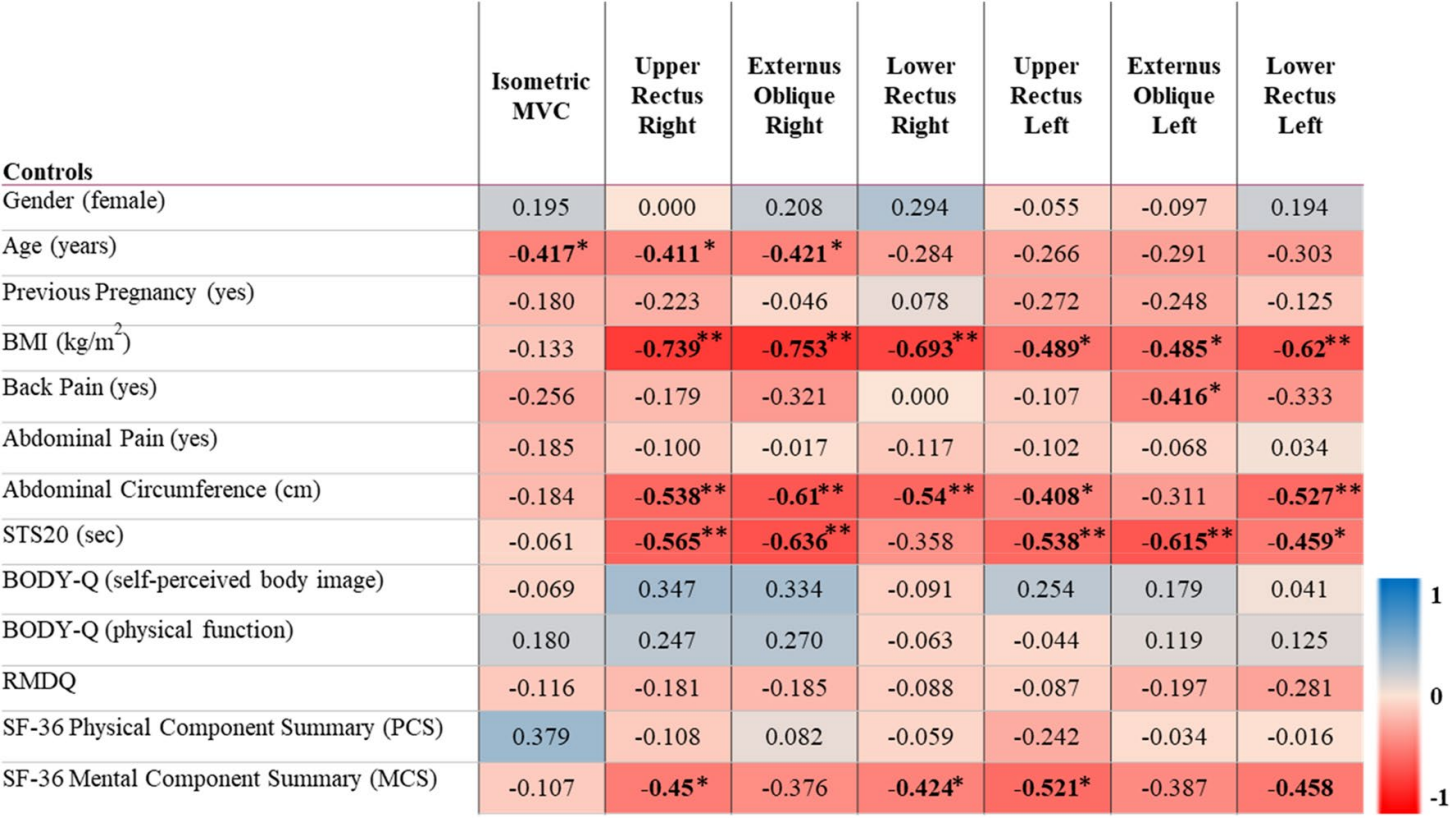
Convergent validity of SEMG amplitude data in control group without ARD

Spearman rank correlations (rho) between mean amplitude of SEMG and other variables. A correlation of 0.5 was considered the smallest correlation of interest. Cut-off values of 0.1–0.29 small, 0.3–0.49 moderate, 0.5–0.69 large, 0.7–0.89 very large and 0.9–1.0 extremely large were used to determine the strength of the correlations (28). To better show the correlations, red represents rho-values − 1 and blue 1

**Correlation is significant at the 0.01 level (2-tailed)

*Correlation is significant at the 0.05 level (2-tailed)

### Recruitment order

Notably, the recruitment order of the abdominal muscles differed between groups. External oblique muscles were the first ones to be contracted in 50% of the cases during isometric abdominal strength tests in patients with ARD, the lower rectus in 32% and upper rectus in 18%. In contrast, among the controls, an almost even distribution of muscular activation (upper rectus, 34%, external obliques 34%, and lower rectus 32%) was observed.

## Discussion

### Relative and absolute reliability

This study shows a moderate to excellent relative test–retest reliability when examining the mean amplitude of SEMG at six specific sites of the abdominal wall during an isometric abdominal muscle strength test in patients with ARD. Similarly, amplitude demonstrated the best relative reliability in the control group, ranging from poor to good. Relative reliability of fatigue measurements was generally poor in both groups, while for recruitment order, the relative reliability was poor to good in both groups.

To our knowledge, only one previous study has used SEMG in patients with ARD. However, that study only included women with ARD postpartum and did not report any data on reliability [31]. In our study, relative reliability was similar between patients with SEMG and among a control group without ARD matched for age, sex, and BMI, thus indicating that the presence of ARD does not seem to impact measurement reliability. Similarly, and even slightly better than our findings on amplitude, very good to excellent relative reliability has been shown with SEMG amplitudes while performing different exercises of the trunk in healthy young participants with normal BMI [32–34].

Adding SEMG during an isometric abdominal strength test is suggested as a useful instrument for objectively assessing abdominal muscle function [32–34]. It can provide valuable information on neuromuscular control of abdominal muscles during specific tasks, valuable in both the research and the clinical setting. In addition, important knowledge can be gained on muscle fatigue and the effects of training or rehabilitation programs on abdominal muscle function. Escamilla et al. [35] evaluated different abdominal exercises with SEMG and concluded that there is a linear relationship between amplitude and isometric abdominal strength test when the muscle length is not changing rapidly, which is also the case in our isometric test setting. Our findings of moderate to excellent relative test–retest reliability for amplitude are thus encouraging. However, it should be noted that the measures of absolute reliability were relatively large, which might impact the ability of these SEMG amplitude measurements to detect changes over time [36]. To clarify this further, the responsiveness of the measurements needs to be determined, which was, however, not within the scope of the current study.

### Convergent validity

While there is a strong correlation between the isometric abdominal wall strength test and self-reported abdominal muscle strength, previous research fails to verify its convergent validity. This is evident in the absence of correlations with relevant measurements in individuals with abdominal rectus diastasis (ARD), including ARD width, body mass index (BMI), abdominal circumference, and daily activity levels [10, 11]. Similarly, our results demonstrate no significant correlation between isometric abdominal strength and the abovementioned measurements nor other relevant measurements, including back pain or functional performance. However, SEMG in combination with an isometric abdominal strength test renders clear added value. For example, SEMG amplitude measurements demonstrated moderate to very large correlations with several measurements, including back pain, abdominal circumference, upper ARD width, self-perceived body imaging, and sit-to-stand performance—measurements that were also significantly different from the control group matched for age, sex, and BMI, indicating clinical relevance.

To our knowledge, this study is the first to determine the convergent validity of SEMG measurements during an isometric abdominal strength test in patients with ARD. Nevertheless, a few previous studies have investigated the validity of SEMG, including Varol et al. [37], who observed moderate associations (r = 0.359) between SEMG-assessed muscle activity (i.e. SEMG slope and ultrasound-measured muscle thickness), and hand grip force (i.e. muscle fatigue assessed by a hand grip dynamometer, where the participants performed a 15 s maximum voluntary isometric contraction (MVIC) of the hand). Their findings are slightly lower than those observed in the present study, which demonstrated moderate to very large correlations. The study by Hills et al. [38] assessed correlations between abdominal muscle endurance (measured with electromagnetic sensors), isometric abdominal strength (with the help of a dynamometer), ARD width, and lumbar pain. They found a significant negative association between abdominal muscle strength (i.e. trunk rotation torque) (r = -0.367) and the ability to perform a sit-up (r = − 0.514). Significant negative correlations were also found by Liaw et al. [8] between the width of the ARD and dynamic (r = − 0.36) and static (r = − 0.42) endurance tasks based on different abdominal crunch exercises, and not isometric strength as used in the present study. Their findings are, therefore, not entirely comparable to ours.

### Recruitment order

Our results indicated a difference in the muscular recruitment pattern in the two groups. Notably, our findings are merely based on descriptive comparisons and are not assessed statistically, since we did not have statistical power for this variable, so the results should be interpreted carefully. Nonetheless, we observed that the ARD group activated the external oblique first in 50% of the cases when performing an isometric abdominal strength test, compared to the control group, where the activation was evenly spread over the three investigated muscle groups. Our findings suggest that patients with ARD might compensate for the weakened rectus muscles by increasing the activation of other muscles, such as the external and internal oblique muscles. It is an interesting new observation that the pattern of activation may diverge between patients with ARD and controls. Difficulties arise when comparing these results to earlier studies, since the latter have investigated healthy young participants without ARD. To the best of our knowledge, studies in which the muscular recruitment pattern is examined in patients with ARD are lacking. Mandroukas et al. [39] studied the order of muscular activation during dynamic and isometric exercises and found an early activation of the external obliques during sit-ups. This contrasts with our finding of evenly distributed activation in the control group. Also, their task was a dynamic movement compared to our isometric abdominal strength test. Another example is a study by Hodges et al. [40], which compares abdominal muscle activation patterns during different arm exercises. They found that the m. transversus abdominis was first recruited in all exercises, and that the rectus abdomini muscles were activated earlier when the spine was more engaged during lifts above the head. We did not examine the transversal muscle since it is difficult to examine without intramuscular EMG and, therefore, it is problematic to compare these outcomes. Taken together, our findings plus previous findings suggest that a deeper analysis of muscular recruitment patterns in patients with ARD could be of interest to understand the change in the abdominal wall and its function after ARD occurs.

### Strengths and limitations

By clearly defining criteria for patients with ARD, we were able to examine and match them with controls without ARD. This improved the study’s ability to draw meaningful conclusions, as did adhering to the GRRAS Guidelines [16]. Furthermore, the procedures, including the isometric abdominal muscle strength test, surface EMG collection, and clinical investigations, were standardized. This standardization promoted consistency and reduced potential sources of variability in data collection. Data accuracy was also enhanced by detailed documentation and by employing one single investigator with the aim of reducing inter-rater bias.

By having a single investigator conduct all tests, we strived to ensure that the same standardized procedures were consistently applied across all participants. This helped minimize variations introduced by different observers, increasing the reliability and internal validity of the study. A range of outcome measures, including clinical investigations, isometric abdominal muscle strength, surface EMG data, and questionnaire responses, provided a comprehensive assessment of the participants' health and functional status.

Regarding limitations of our study, its manual measurement of ARD could be criticized. Several methods exist to measure the width of ARD, and a systematic review by van der Water et al. [41] showed that ultrasound can perhaps give a more correct measurement compared to palpation and measuring tape, which can overestimate the diastasis. On the other hand, Emanuelsson et al. [42] showed that a measuring tape is more correct than computer tomography when comparing measurements during surgery. Several of the participants did not have their widest diastasis at the midpoint level. Instead, the diastasis was widest just above and below the umbilicus. Mota et al. [43] support this observation, showing that women had their widest diastasis 2 cm above and below the umbilicus post-partum. Also, the selection of participants must be taken into consideration. The participants in our study were unselected and required help for bodily pain and functional impairments in daily life. However, there was not one particular symptom considered essential. They scored lower than the control group when asked about quality of life and self-perceived body image. ARD can have significant psychological and social repercussions, contributing to lower self-esteem, and an overall decrease in quality of life as seen both in previous work from our group [6, 21] and Nielsen et al. [44] where patients scored lower in their quality-of-life variables compared to controls.

Secondly, the limitations of measuring isometric abdominal strength with a computerized dynamometer should be highlighted. Even if Stark et al. [11] and Gunnarsson et al. [9] showed that isometric muscle strength measured on a computerized dynamometer is a reliable tool to examine the abdominal muscles, a possible activation of ilio-psoas and femoral muscles cannot be excluded, since the movement cannot isolate the contraction to the abdominal muscles. Escamilla et al. [35] showed different effects of rectus femoris on the classic abdominal crunch and the bent-knee sit-up with the legs fixated. If only the legs are fixated, a significant larger portion of rectus femoris would be activated. Compared to this arrangement, the rectus abdominis and the obliques were recruited earlier and more strongly in our study since both the trunk and legs were fixated.

Thirdly, SEMG captures signals from more than one muscle fiber, and recordings might be influenced by individual variations of subcutaneous layers and skin quality [45]. These facts should be kept in mind when interpreting data. Otherwise, SEMG is non-invasive and thus not harmful to the participants.

Lastly, the selection of SEMG variables to be studied was made in line with observations by Monfort Pañego et al. [46]. The potential risk of crosstalk, which can occur when signals from adjacent muscles are picked up by the electrodes, should therefore be mentioned [47]. We are aware of potential sources of bias and have taken steps to minimize their impact on the data to ensure accurate and meaningful interpretations of the results. In our study, we have focused on the mean amplitude of the five contractions during isometric abdominal strength tests, where one single investigator processed all signals. Chowdhury et al. [48] discuss in their review how to handle the filtering and interference of random noises from EMG signals and the importance of consistency by the individual that filters the recorded signals in reducing the risk of bias.

### Clinical implications

Adding SEMG amplitude measurements in an isometric strength test appears valuable when comparing muscle activity in patients with abdominal rectus diastasis (ARD) and might also be relevant for evaluating changes prior to and after surgery. In forthcoming studies, adding SEMG to an isometric strength test can potentially also be used to objectively assess the efficiency of surgery as a treatment method. These studies can also demonstrate if the results are sensitive to changes over time or with longer intervals between tests, and potentially also identify patients who would benefit from surgery or other interventions. Additionally, the moderate to strong correlations showed that in patients with ARD, lower SEMG amplitudes are associated with more symptoms of back pain, longer functional sit-to-stand test durations, and lower health-related quality-of-life and body perception. These correlations are not observed with isometric abdominal muscle testing alone, making SEMG measurements a valuable source of additional information concerning patients and their problems.

## Conclusions

SEMG amplitude measurements during isometric abdominal wall strength testing demonstrate moderate to excellent relative reliability with acceptable but relatively large absolute reliability among patients with ARD and age and sex-matched controls. Importantly, and in contrast to performing an isometric abdominal wall strength test alone, adding a measurement of SEMG amplitude provides additional value as evidenced by moderate to strong correlations with BMI, back pain, abdominal circumference, upper ARD width, self-perceived body imaging, and sit-to-stand performance, supporting the convergent validity. Future steps, preferably with a larger sample size, include determining the responsiveness of these measurements to standard exercise training or reconstructive abdominal wall procedures. The authors declare no conflict of interest. The Declaration of Helsinki principles of ethical standards were followed, and written informed consents were obtained. The study was approved by the Regional Ethical Review Board (Dnr: 2019-05348, 2020-04335). Note regarding Body-Q: "The content and design of the questionnaire are protected by U.S. and international intellectual property laws, and use was made under a license from Memorial Sloan Kettering Cancer Center, New York, NY, USA. 

## Data Availability

No, our records are not in a repository that is accessable for the moment.
